# Comparative Analysis of Laparoscopic Gastrectomy Versus Laparoscopic-Assisted Gastrectomy: Postoperative Complications

**DOI:** 10.7759/cureus.51186

**Published:** 2023-12-27

**Authors:** Iván Josué Calderón-Canseco, Claudia B Domínguez-Fonseca, Militza Cerrillo-Miranda, Manuel A Pérez-Turrent, Sonia Fernández-Ananín, Eduardo María Targarona Soler, María Carmen Balagué-Ponz

**Affiliations:** 1 General Surgery, Hospital General Regional No. 1 "Dr. Carlos Mac Gregor Sanchez Navarro" Instituto Mexicano del Seguro Social, Mexico City, MEX; 2 Gastrointestinal and Hematological Surgical Unit, Hospital de la Santa Creu I Sant Pau, Autonomous University of Barcelona, Barcelona, ESP

**Keywords:** partial gastrectomy, total gastrectomy, hand-assisted laparoscopic gastrectomy, laparoscopic gastrectomy, gastric cancer

## Abstract

The evolution of laparoscopic surgery in gastric cancer has advanced significantly, with benefits over open surgery initially demonstrated in early gastric cancer and later in advanced stages. This study aims to evaluate postoperative complications, surgical outcomes, and anastomosis safety by comparing laparoscopic gastrectomy and laparoscopic-assisted gastrectomy. This retrospective, observational, analytical study included patients diagnosed with gastric cancer who underwent laparoscopic gastrectomy at a university hospital from January 2006 to February 2018. Patients were initially divided into two groups based on the type of anastomosis: laparoscopic gastrectomy (intracorporeal anastomosis) and laparoscopic-assisted gastrectomy (extracorporeal anastomosis). Further secondary analysis was done with subgroups based on the type of gastrectomy and anastomosis performed. A total of 139 patients were analyzed, showing significant differences in postoperative complications between the two surgical approaches. The laparoscopic-assisted group exhibited a higher rate of complications. The laparoscopic approach (with intracorporeal anastomosis) was found to have a lower risk of complications and morbidity/mortality compared to the laparoscopic-assisted approach. Laparoscopic gastrectomy with intracorporeal anastomosis resulted in lower morbidity and mortality than laparoscopic-assisted gastrectomy. The technique of partial gastrectomy with intracorporeal anastomosis was associated with the lowest rate of postoperative complications.

## Introduction

Gastric cancer is the fifth most commonly diagnosed tumor and stands as the fourth leading cause of cancer-related mortality as per the World Health Organization [1]. Within our local setting, it manifests with a prevalence of 9.93 per 100,000 individuals [2], and an overall five-year survival rate remaining low, not surpassing 25% [3]. Laparoscopic gastrectomy is advocated among current therapeutic modalities, owing to its pronounced benefits in pain management and recovery time [4].

The first laparoscopic-assisted distal gastrectomy for gastric cancer was described by Kitano et al. in 1994 [5]. The evolution of laparoscopic surgical interventions for gastric cancer has been substantial, driven by an array of technical enhancements and the substantiated superiority over open surgical techniques. These benefits were initially observed in early gastric cancer and, subsequently, several authors have also reported favorable outcomes in advanced cancer. The laparoscopic approach initially entailed an assisted approach, utilizing a mini-laparotomy for anastomotic construction (laparoscopically assisted). However, the accrued expertise in intracorporeal anastomosis within the domain of bariatric surgery has aided the technical evolution of surgical groups dedicated to gastric surgery.

The advancement of bariatric surgery has set a paradigm for teaching laparoscopic surgical techniques, mirroring numerous technical maneuvers utilized in other interventions [6]. This discipline facilitates the development of experience in crafting high anastomoses combining the use of staplers and intracorporeal suturing, as well as navigating challenging visual fields. All these aspects aid the learning curve in laparoscopic surgery for gastric cancer [7].

Partial or total gastrectomy can be performed via a laparoscopic approach or may include an assisted phase. Among these, the greatest challenge lies in total laparoscopic gastrectomy, characterized by a high intracorporeal esophagojejunostomy anastomosis. In the laparoscopically assisted approach, this anastomosis is performed through a 5-7 cm incision situated in the upper midline of the abdomen. In certain cases, especially in the obese population or when faced with a short esophageal stump, it becomes imperative to extend the incision to ensure a safe anastomosis [8].

Given the absence of preexisting studies detailing the laparoscopic management of gastric cancer in our region, we undertook an epidemiological analysis of this pathology at our institution, a tertiary-level referral hospital serving a health area of 470,000 individuals. The aim of this study was to evaluate postoperative complications, post-surgical evolution, and the safety of the anastomosis by comparing laparoscopic gastrectomy and laparoscopic-assisted gastrectomy.

## Materials and methods

This retrospective analytical observational study included patients diagnosed with gastric cancer who underwent laparoscopic gastrectomy at a university-affiliated hospital, Hospital de la Santa Creu i Sant Pau in Barcelona, Spain, from January 2006 through February 2018. As of 2014, this data pool has been incorporated into the European Registration of Cancer Care (EURECCA) Upper GI Group registry. This study was approved by the Institutional Review Board and Ethical Committee for Medical Research of the Fundación de Gestió Sanitària del Hospital de la Santa Creu i Sant Pau of Barcelona, Spain (approval number: IIBSP-GAS-2018-41).

For initial analytical purposes, the patients were bifurcated into two cohorts based on the anastomotic technique employed: one consisting of patients undergoing laparoscopic gastrectomy with intracorporeal anastomosis (LG) and the other comprising those who had laparoscopic-assisted gastrectomy with extracorporeal anastomosis (AG). A detailed secondary analysis ensued, stratifying these patients into subgroups predicated upon the specific gastrectomy executed-total gastrectomy (TG), partial gastrectomy (PG), assisted total gastrectomy (ATG), and assisted partial gastrectomy (APG).

Prior to surgical intervention, all patients were subjected to a comprehensive preoperative diagnostic regimen, including gastroscopy, histological evaluation, thoraco-abdomino-pelvic CT for extension assessment, and standard preoperative studies. We compiled a suite of variables (epidemiological, perioperative, and histological) extending up to 30 days postoperatively or until the patient's discharge from the hospital.

The histological assessment adhered to the classification system developed by Nakamura and Sugano [9]. Tumoral staging was conducted in alignment with the criteria set forth by the American Joint Committee on Cancer (AJCC) and utilized the TNM (tumor, node, metastasis) classification as revised in 2009 [10]. Postoperative complications were categorized based on the Clavien-Dindo grading system [11].

The statistical analysis was conducted using IBM SPSS Statistics for Windows, Version 25.0 (Released 2017; IBM Corp; Armonk, New York, United States). Categorical variables are reported as relative frequencies and as absolute counts. Quantitative variables are articulated as arithmetic means and standard deviations. The chi-squared test or Fisher's exact test was employed for the comparison of proportions. For mean comparisons, depending on the data distribution, the Mann-Whitney U test, Kruskal-Wallis test, Student's t-test, or ANOVA were applied. A significance threshold was set at the 5% level (p ≤ 0.05).

## Results

Data from 139 patients who underwent surgery for gastric cancer were analyzed: 74 cases were in the LG group and 65 cases were in the AG group. Of the 74 cases in the LG group, 17 were TGs and 57 were PGs, while in the AG group, there were 27 TGs and 38 PGs.

When examining the epidemiological characteristics, no significant differences were found in terms of age, gender, body mass index, or anesthetic risk as assessed by the American Society of Anesthesiologists classification and the histological study also showed no differences between the two groups (Tables 1, 2)

**Table 1:** Epidemiological characteristics LG: laparoscopic gastrectomy with intracorporeal anastomosis; AG: laparoscopic-assisted gastrectomy with extracorporeal anastomosis; BMI: body mass index

		LG (n = 74)	AG (n = 65)	p-value (≤ 0.05)
Age (years), mean ± SD		71 ± 11.1	70.6 ± 10.6	0.641
Sex, n (%)	Male	40 (54.1%)	40 (61.5%)	0.395
	Female	34 (45.9%)	25 (38.5%)	
BMI (kg/m^2^), mean ± SD		25.4 ± 4.4	25.1 ± 4.4	0.707
ASA Clasification, n (%)	I	3 (4.1%)	2 (3.1%)	0.847
	II	25 (33.8%)	25 (38.5%)	
	III	42 (56.8%)	33 (50.8%)	
	IV	4 (5.4%)	5 (7.7%)	

**Table 2:** Histological characteristics LG: laparoscopic gastrectomy with intracorporeal anastomosis; AG: laparoscopic-assisted gastrectomy with extracorporeal anastomosis

		LG (n = 74)	AG (n = 65)	p-value (≤ 0.05)
Tumor localization, n (%)	Antrum	49 (66.2%)	33 (50.8%)	0.175
	Body	18 (24.3%)	24 (36.9%)	
	Fundus	7 (9.5%)	8 (12.3%)	
Histology, n (%)	Differentiated	41 (55.4%)	41 (63.1%)	0.391
	Not differentiated	33 (44.6%)	24 (36.9%)	
TNM Stage, n (%)	0	2 (2.7%)	1 (1.5%)	0.502
	IA	16 (21.6%)	10 (15.4%)	
	IB	10 (13.5%)	14 (21.5%)	
	IIA	14 (18.9%)	18 (27.7%)	
	IIB	10 (13.5%)	6 (9.2%)	
	IIIA	10 (13.5%)	6 (9.2%)	
	IIIB	5 (6.8%)	7 (10.8%)	
	IIIC	7 (9.5%)	3 (4.5%)	
Size of tumor (cm), mean±SD		5.2 ± 3.3	5.7 ± 3.1	0.403
Lymphadenectomy, n (%)	D1	20 (27.0%)	14 (21.5%)	0.554
	D2	54 (72.0%)	51 (78.5%)	
Number of resected lymph nodes, mean±SD		30.5 ± 16.2	25.8 ± 14.8	0.083
Positive lymph nodes, mean±SD		4.3 ± 7.0	4.4 ± 6.9	0.968

Postoperative outcomes were evaluated considering the following variables: initiation of diet, in-hospital stay, surgical complications, medical complications, reoperation rate, and mortality (Table 3).

**Table 3:** Postoperative outcomes LG: laparoscopic gastrectomy with intracorporeal anastomosis; AG: laparoscopic-assisted gastrectomy with extracorporeal anastomosis

	LG (n= 74)	AG (n= 65)	p-value (≤ 0.05)
Start of diet (days), mean±SD	4.1 ± 2.6	5.1 ± 2.6	0.036
Hospitalization days, mean±SD	11.5, ± 12.0	17.1 ± 11.6	0.005
Reintervention, n (%)	5 (6.8%)	11 (16.9%)	0.069
Mortality, n (%)	0 (0.0%)	7 (10.8%)	0.004
Surgical complications, n (%)	15 (20.2%)	27 (41.5%)	0.038
Medical complications, n (%)	10 (13.5%)	5 (7.7%)	0.412

The initiation of diet and the duration of in-hospital stay were significantly shorter in the LG group compared to the AG group. Surgical postoperative complications, as well as 30-day mortality, were significantly higher in the AG group.

Table 4 details all postoperative complications, with anastomotic dehiscence (gastro-jejunal or esophago-jejunal) showing a statistically significant difference.

**Table 4:** Postsurgical complications LG: laparoscopic gastrectomy with intracorporeal anastomosis; AG: laparoscopic-assisted gastrectomy with extracorporeal anastomosis Data given as n (%)

-	LG (n= 74)	AG (n= 65)	p-value (≤ 0.05)
Total	25 (33.8%)	32 (49.2%)	0.084
Surgical complications	15 (60%)	27 (84%)	0.038
Dehiscence of esophageal-jejunal or gastrojejunal anastomosis	4 (26.67%)	11 (40.7%)	0.029
Deshiscense of Roux-en-Y jejunal-jejunal anastomosis	0 (0.0%)	2 (7.4%)	0.217
Dehiscence of duodenal stump	1 (6.67%)	1 (3.7%)	1.000
Jejunostomy occlusion	1 (6.67%)	0 (0.0%)	1.000
Hemorrhage with transfusional requirement	0 (0.0%)	1 (3.7%)	0.468
Abscess	2 (13.34%)	5 (18.5%)	0.252
Intestinal perforation	0 (0.0%)	1 (3.7%)	0.468
Ileus	5 (33.34%)	3 (11.1%)	0.723
Pancreatic fistula	1 (6.67%)	0 (0.0%)	1.000
Evisceration	0 (0.0%)	1 (3.7%)	0.468
Transhiatal internal hernia	0 (0.0%)	1 (3.7%)	0.468
Wound infection	1 (6.67%)	1 (3.7%)	1.000
Medical complications	10 (40%)	5 (16%)	0.412
Catheter infection	2 (20%)	4 (80%)	0.418
Urinary Infection	2 (20%)	1 (20%)	1.000
Respiratory infection	4 (40%)	0 (0.0%)	0.123
Pneumothorax	1 (10%)	0 (0.0%)	1.000
Acute kidney injury	1 (10%)	0 (0.0%)	1.000

Table 5 displays the Clavien-Dindo classification of these complications. The majority of complications were classified as type II and IIIA, which required pharmacological, radiological, or endoscopic management, and thus were considered mild. However, the analysis clearly shows a difference in the grade V complications, which corresponded to death.

**Table 5:** Clavien-Dindo classification LG: laparoscopic gastrectomy with intracorporeal anastomosis; AG: laparoscopic-assisted gastrectomy with extracorporeal anastomosis

	LG (n= 74)	AG (n= 65)	p-value (≤ 0.05)
I	0 (0.0%)	0 (0.0%)	0.040
II	11 (44.0%)	8 (25.0%)	
IIIA	8 (32.0%)	6 (18.8%)	
IIIB	2 (8.0%)	2 (6.3%)	
IVA	1 (4.0%)	3 (9.4%)	
IVB	3 (12.0%)	6 (18.0%)	
V	0 (0.0%)	7 (21.9%	

Statistically significant differences were observed in the number of hospital stay days, with a longer duration in the ATG group. Similarly, mortality was higher in the assisted gastrectomy groups (both ATG and APG) with statistical significance (Table 6).

**Table 6:** Subgroup results TG: total laparoscopic gastrectomy; ATG: assisted total gastrectomy; PG: partial gastrectomy; APG: assisted partial gastrectomy

	TG (n = 17)	ATG (n = 27)	PG (n = 57)	APG (n = 38)	p-value (≤ 0.05)
Start of diet (days), mean±SD	6 ± 2.4	5.2 ± 2.6	3.6 ± 2.6	5.1 ± 2.6	0.003
Hospitalization days, mean±SD	15.7 ± 11.3	20.4 ± 11.9	10.3 ± 12.0	14.7 ± 11.6	0.002
Reintervention, n (%)	3 (17.6%)	6 (22.2%)	2 (3.5%)	5 (13.2%)	0.061
Mortality, n (%)	0 (0.0%)	4 (14.8%)	0 (0.0%)	3 (7.3%)	0.019
Total complications	8 (47.1%)	16 (59.3%)	17 (29.8%)	16 (42.1%)	0.074
Surgical complications	6 (35.2%)	13 (48.1%)	9 (15.7%)	14 (36.8%)	0.013
Anastomosis dehiscence	3 (17.6%)	7 (25.9%)	1 (1.8%)	4 (10.5%)	0.007
Medical complications	2 (11.8%)	3 (11.1%)	8 (14.0%)	2 (5.3%)	0.604

Regarding the postoperative complications of the four groups, a significantly higher incidence of postoperative complications was found in patients who underwent assisted total gastrectomy. This statistically significant difference was due to anastomotic dehiscence.

## Discussion

This cohort of 139 patients appears adequate to provide indicative results when assessing these technical aspects, although the numbers are much lower compared to some Asian studies [8,12-14].

Regarding laparoscopic technique, it has been established that competence is attained after performing 40 intracorporeal anastomoses [15]. It might be assumed that the assisted technique is chosen due to the difficulty of performing the esophagojejunal anastomosis with an intracorporeal suture. However, our study revealed that the assisted laparoscopic technique had a higher incidence of complications. This fact is not solely justified by it corresponding to the initial part of the series, thus including a larger percentage of the learning curve. It is likely also associated with greater difficulty in executing the high anastomosis due to the need to work through a reduced incision and at a greater distance from the anastomosis site, which results in increased tissue traction and visual difficulties. In contrast, laparoscopic surgery (with intracorporeal anastomosis) presented a lower risk of complications and lower morbidity and mortality, for both PGs and TGs.

At our center, as at many others in our region, bariatric surgery is performed by the same team that carries out esophagogastric surgery. The expansion of bariatric surgery has facilitated improved skills in performing laparoscopic intracorporeal sutures, achieving the necessary dexterity. We believe that the experience gained at our center with bariatric surgery has allowed us to acquire this expertise and progress to performing intracorporeal anastomoses.

From an oncological perspective, the surgical specimens are comparable to those obtained in open surgery, and no significant differences were observed when comparing both laparoscopic approaches, although lymph node resection was slightly higher in laparoscopic than in assisted gastrectomies. In 82% of all surgeries, more than 25 lymph nodes were resected. The removal of a sufficient number of nodes has been shown to allow better tumor staging and to improve the therapeutic approach and prognosis of the patient [10,16]. However, this is not the only parameter to suggest that laparoscopic management of gastric cancer is appropriate and feasible.

Laparoscopic gastrectomy for the curative treatment of gastric cancer is associated with high rates of postoperative complications [17]. In Spain, Escrig et al. report medical complications in 24% and surgical complications in 29.4% postoperatively [18], while Climent et al. report an overall complication rate of 61% postoperatively [17]. In our series, the prevalence of postoperative complications is 41%, with medical complications occurring in 10.7% and surgical complications in 30.3%, being higher in laparoscopic-assisted gastrectomies. These complication rates remain higher than those reported in Asian series [8,12-14]. Although we acknowledge that screening, patient characteristics, and experience in these centers are not comparable with Western series. The ranges of global postoperative morbidity reported in the literature are very broad and largely depend on the thoroughness of complication recording [19]. Moreover, we must consider that the average age of our patients was over 70 years, with more than 60% classified as ASA III-IV, which could justify a higher rate of morbidity and mortality. The older age of our study population could also justify the higher incidence of cancer in the gastric body-antrum.

Regarding postoperative mortality, our series showed a rate of 5%, which was higher in laparoscopic-assisted gastrectomy, 10.8%. We believe that the increased mortality observed in the AG group can be attributed to the learning curve and expertise required for performing a total laparoscopic gastrectomy. Although all our procedures are now performed laparoscopically, this retrospective non-experimental study revealed higher mortality rates at the study's outset, in contrast to the recent cases completed entirely through laparoscopy. As mentioned before, the mortality associated with this disease exhibits significant variability, and different series from several Western European countries report a postoperative mortality rate exceeding 5% after gastrectomy [3,17,18].

The present study has a number of limitations. The results should be interpreted taking into account the limitations of our retrospective design and its 12-year study period (2006-2018). Similarly, it should be considered that cases with intracorporeal anastomosis are more recent compared to those performed with extracorporeal anastomosis, and consequently, the learning curve is predominantly included in the latter group. This may partly justify the differences in the incidence of complications, although, as we referred to at the beginning of the discussion, it is not the only justification for the results since there are technical aspects to consider.

## Conclusions

This study underscores the importance of surgical experience, particularly gained through performing bariatric surgery, in achieving proficiency with laparoscopic intracorporeal sutures. Our results demonstrate that laparoscopic approaches yield surgical specimens comparable to open surgery, with slightly higher lymph node resection in laparoscopic gastrectomies. However, the benefits extend beyond comparable oncological outcomes, as laparoscopic management allows for better tumor staging. Despite high postoperative complication rates, our findings suggest that laparoscopic gastrectomies, particularly with intracorporeal anastomoses, exhibit lower morbidity, lower postoperative complications, and mortality than their assisted counterparts.
